# Dietary Supplements and Natural Products: An Update on Their Clinical Effectiveness and Molecular Mechanisms of Action During Accelerated Biological Aging

**DOI:** 10.3389/fgene.2022.880421

**Published:** 2022-04-28

**Authors:** Ye Chen, Sherif Hamidu, Xintong Yang, Yiqi Yan, Qilong Wang, Lin Li, Patrick Kwabena Oduro, Yuhong Li

**Affiliations:** ^1^ State Key Laboratory of Pharmacology of Modern Chinese Medicine, Department of Pharmacology and Toxicology, Institute of Traditional Chinese Medicine, Tianjin University of Traditional Chinese Medicine, Tianjin, China; ^2^ Clinical Pathology Department, Noguchi Memorial Institute for Medical Research, University of Ghana, Legon, Ghana

**Keywords:** aging, dietary supplements, natural products, mitochondrial dysfunction, age-related diseases, nutrient-sensing pathway

## Abstract

Accelerated biological aging, which involves the gradual decline of organ or tissue functions and the distortion of physiological processes, underlies several human diseases. Away from the earlier free radical concept, telomere attrition, cellular senescence, proteostasis loss, mitochondrial dysfunction, stem cell exhaustion, and epigenetic and genomic alterations have emerged as biological hallmarks of aging. Moreover, nutrient-sensing metabolic pathways are critical to an organism’s ability to sense and respond to nutrient levels. Pharmaceutical, genetic, and nutritional interventions reverting physiological declines by targeting nutrient-sensing metabolic pathways can promote healthy aging and increase lifespan. On this basis, biological aging hallmarks and nutrient-sensing dependent and independent pathways represent evolving drug targets for many age-linked diseases. Here, we discuss and update the scientific community on contemporary advances in how dietary supplements and natural products beneficially revert accelerated biological aging processes to retrograde human aging and age-dependent human diseases, both from the clinical and preclinical studies point-of-view. Overall, our review suggests that dietary/natural products increase healthspan—rather than lifespan—effectively minimizing the period of frailty at the end of life. However, real-world setting clinical trials and basic studies on dietary supplements and natural products are further required to decisively demonstrate whether dietary/natural products could promote human lifespan.

## Graphical Abstract

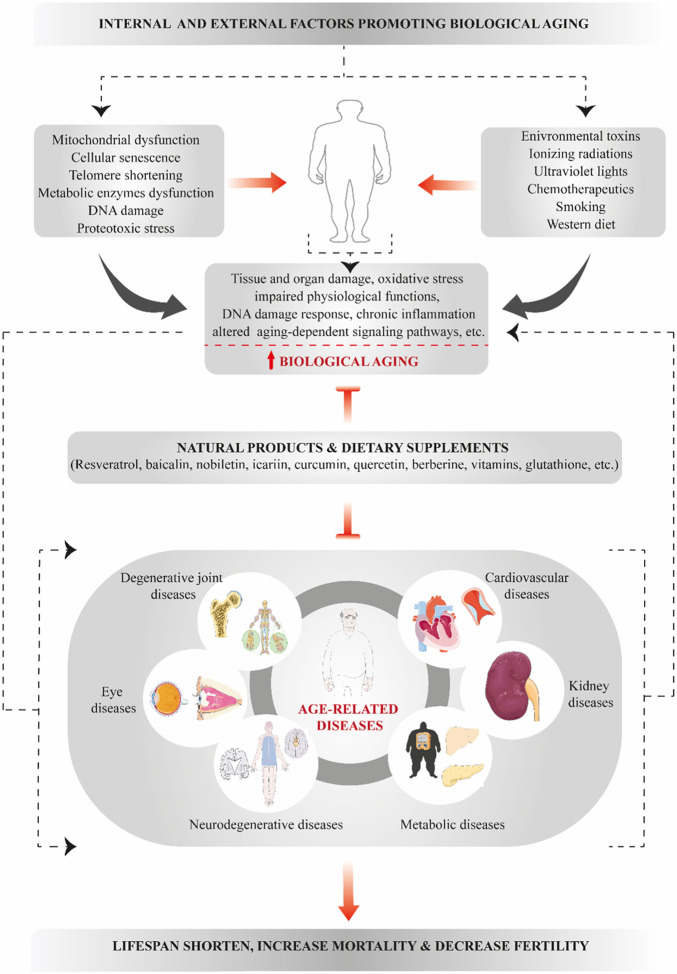

## 1 Introduction

Accelerated biological aging is an unfavorable condition characterized by the slow deterioration in physical and biological functions, which is accompanied by high multimorbidity, mortality, disability, and low fertility rates. According to the World Population Aging Report by the United Nations, the world is witnessing a profound growth in the number and proportion of older people aged 65 and over. Moreover, it is projected that by 2050, the aged population will have doubled to 1.5 billion worldwide. However, despite the continual expected increase in the aging population, the good news is that there is substantial improvement in the geriatric population’s life expectancy and survival (United Nations Department of Economic and Social Affairs, 2019).

**FIGURE 1:**
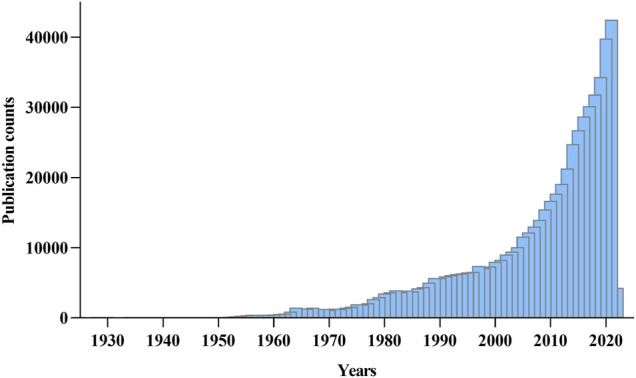
Yearly growth of aging research across biomedical and medical fields. The number of research publications was viewed from PubMed on 1st February 2022. The search term was “Aging” and “Ageing.”

Although aging is not a disease per se, it is a significant shared risk driver for every major cause of death, disease, and disability compared to sedentary lifestyle activities and obesity (Qian and Liu, 2018; Stekovic et al., 2019). Because as an individual gets old, physiological functions decline, contributing to when and how one gets sick and passes on. Despite this apparent connection, in earlier years, aging was thought to be unmodifiable, but subsequent discoveries point to a change in thinking and suggest that there is a chance to promote longevity and reduce the occurrence of age-related health conditions. Today, scientists have recognized the impact of slowing aging processes, causing aging biology and age-related health conditions to receive the needed attention across all bio and medical research fields (Figure 1).

Scientists have expounded on aging biology since discovering single-gene mutation that doubles life in worms and have identified classic molecular aging hallmarks. These hallmarks of aging include mitochondrial dysfunction, proteostasis loss, cellular communication breakdown, telomere attrition, stem cell exhaustion, cellular senescence, and epigenetic and genomic alterations. Interestingly, evidence indicates that pharmacological interventions can affect the aging process and its hallmarks, raising the possibility of slowing aging and promoting healthy aging.

Dietary supplements and natural products, consisting of various products and ingredients from herbal, marine, and botanical sources, continue to receive remarkable patronage from consumers worldwide. Dietary supplements and natural products have a fascinating history concerning their health benefits in human life and diseases before the birth of western drugs. To ensure health safety in the population, several countries’ Food and Drugs Administrations (FDA) and international health institutions have issued numerous guidelines on dietary supplements and natural product medical applications. Although most regulatory authorities prohibit dietary supplements and natural products from diagnosing, mitigating, treating, curing, or preventing human diseases, a greater pool of FDA-approved drugs is optimized from natural products (Newman and Cragg, 2020).

In aging research, several natural products and dietary supplements continue to be studied extensively for their anti-aging properties or are used as research tools to explore and discover molecular mechanisms underpinning biological aging (McCubrey et al., 2017; Ning et al., 2018; Zhang et al., 2019a; Lin et al., 2019). Moreover, in today’s world, the connection between aging, human aging-related diseases, diet, and natural products has become particularly consequential in the elderly community. Most aged individuals with or without aging-related diseases, such as diabetes type 2, cardiovascular diseases, and cancer, prefer to use natural products or dietary supplements to control or suppress any aging-associated discomforts. This observation has been shown in laboratory studies that nutritional health supplements and natural products reverse or delay age-associated physiological functional declines. And that some of these natural products target essential caloric restriction signaling molecules such as the mammalian target of rapamycin (mTOR), AMP-activated protein kinase (AMPK), Sirtuin 1 (SIRT1), etc., to slow the aging process and age-associated diseases (Sathaye et al., 2017; Wang et al., 2019; Li et al., 2020; Lu et al., 2021; Richardson et al., 2021; Zanco et al., 2021). Thus, understanding the pharmacological intervention of natural and dietary products may hold the promise of extending lifespan and slowing biological aging.

Here, we primarily focus, update, and limit discussion to clinical and preclinical experiments on promising dietary supplements and natural products with effects of slowing accelerated biological aging and age-related diseases. Nevertheless, before this, we will succinctly introduce the fundamental concepts and theories of aging biology and provide an intimate connection between aging and human diseases. We will also briefly discuss some evolutionarily conserved biological aging hallmarks and nutrient restriction-dependent signaling pathways. We look forward to the day when, in the future, this piece will revolutionize thoughts about some dietary supplements and natural products as potential anti-aging agents that can impact human health quality and longevity.

## 2 Aging and Aging-Related Diseases

Human aging is a complex, multidimensional process, perhaps an inevitable or evitable event. It is a well-established significant risk factor that increases vulnerability to human diseases, especially within the community of older adults (Hou et al., 2019). The functional and biological declines witnessed as we age remarkably impact body system processes, switching the balance to massive cellular alterations and organ damages leading to diseases. For instance, as we age, there is a steady drop in the immune system’s ability to execute its intended duties, such as clearing pathogenic insults and establishing long-term immunological memory. This deterioration of the body’s immune system contributes to the occurrence of rheumatoid arthritis, making this disease an aging-associated disease (Boots et al., 2013). In addition, aging causes telomeres to shorten in dimension. Studies show that in rheumatoid arthritis patients, shorter telomeres are frequently seen and may be a causal factor in rheumatoid arthritis development (Heba et al., 2021). In elderly individuals, assessment of their brain function and brain nerves shows unusual features, including cognitive impairment, neuroinflammation, neuron loss, β-amyloid accumulation, the collapse of proteostasis networks, and others. These unusual features are believed to be the root cause of neurodegenerative diseases, particularly Alzheimer’s disease and Parkinson’s disease. This explains why Alzheimer’s disease and Parkinson’s disease are the two most widely studied aging-associated neurodegenerative diseases in the elderly (Coskun et al., 2004; Bender et al., 2006; Ascherio and Schwarzschild, 2016; Hou et al., 2019). The mitochondrion is a metabolic organelle, and it connects with several metabolic signaling mediators. Most of these metabolic signaling mediators, such as SIRT1, AMPK, NAD^+^, and mTOR, are considered longevity mediators because they regulate an organism’s ability to sense and respond to nutrients to increase lifespan. Breakdown in mitochondrial function and metabolic signaling of the above-stated longevity mediators typically happens with time. This breakdown disrupts metabolic health to initiate or progress cardiovascular and metabolic disorders in the elderly (North and Sinclair, 2012; Kane and Sinclair, 2018; Evans et al., 2020a). Cancer is a multifactorial disease with non-specific age-occurrence dynamics (Torre et al., 2016). However, the destruction of cellular and tissue homeostatic processes due to progressive aging subvert immune system machinery and foster the uncontrolled growth of cells (Bottazzi et al., 2018). In addition, insulin/insulin-like growth factor 1 signaling (IIS) mediates longevity; however, cancerous cells expropriate this pathway to aid in uncontrolled proliferation. In all, aging is strongly associated with numerous diseases, and yearly, millions, if not billions, of people are impacted by aging-related diseases. This indicates that if the complete mechanism-based prevention of physiological declines is understood, diseases burden will reduce substantially. Interventions, particularly the anti-aging supplement market, have vital roles to play in this (vide infra), and thus, natural product and dietary supplement impact should not be discounted in the fight against aging.

## 3 The Biological Aging Processes: From Old to New

**FIGURE 2:**
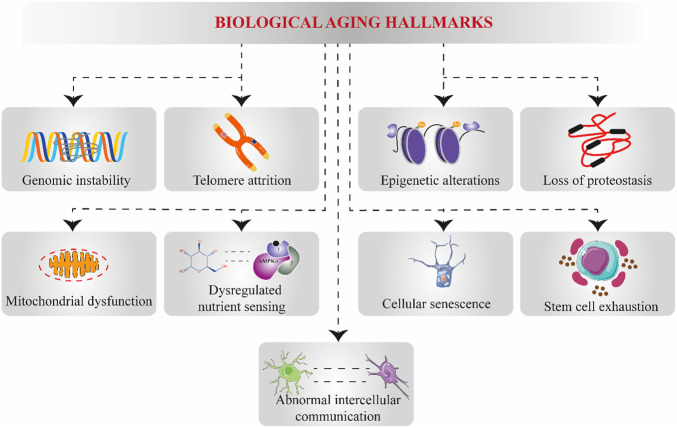
Systematic representation of causal hallmarks underlying biological aging. Biological aging results from multifaceted cellular alterations and breakdowns, which are commonly referred to as “aging hallmarks.” These aging hallmarks include genomic instability, telomere attrition, epigenetic alterations, proteostasis loss, mitochondrial dysfunction, dysregulated nutrient-sensing pathways, cellular senescence, stem cell exhaustion, and abnormal intercellular communication.

First, the process of biological aging involves the impairment or decline of both adaptive and internal homeostatic processes caused by genetic, environmental, and behavioral factors. The mainstream postulated biological aging theories revolve around stress and stress-buffering mechanisms impacting interrelated metabolic signaling pathways (Bektas et al., 2018). The free radical theory of aging once remained the most widely known theory underpinning the primary aging process (Balaban et al., 2005; Finkel and HolbrookOxidants, 2000). Nevertheless, in 1988 and 1993, single-gene mutations discovered in *Caenorhabditis elegans* were associated with a remarkably long lifespan. In addition, behavioral modifications can also influence aging, such as caloric restriction, stress, infection exposure, and pharmaceutical and dietary interventions. All these factors can alter how long someone lives (lifespan) and how long they remain healthy (healthspan). Over the past three decades, these discoveries have paved the way for aging biology and raised the tantalizing possibility that lifespan and healthspan can be extended and accelerated aging processes can be slowed. The quest to characterize the biological underpinnings of aging by scientists has led to identifying what is commonly referred to as “hallmarks of aging (Figure 2).” Although generally, it remains unclear yet how these “hallmarks of aging” might be accelerating biological aging, most of these “hallmarks of aging” are linked and implicated in cellular and tissue metabolic dysfunctions and have also been used to measure cellular aging (Rattan, 2006; Simpson et al., 2016; Canudas et al., 2019). Thus, a deeper understanding of the biology of “hallmarks of aging” and their effects on disease susceptibility could lead to pharmaceutical and dietary interventions to slow the aging process. In fact, senolytic drugs (small molecule compounds that selectively eradicate or destroy senescent cells) and senomorphic drugs (small molecules that interfere with the biological process of senescence by either suppressing the formation of senescent cells or senescence-associated secretory phenotype) are currently being investigated, as part of strategies to slow biological aging and increase healthspan (Paez‐Ribes et al., 2019; Martel et al., 2020). Because of our review focus, we will delve a bit deeper into mitochondrial dysfunction and metabolic and nutrient-sensing node pathways. Later, we will show how natural products and dietary supplements interventions target mitochondria and nutrient-sensing genes to influence an organism’s ability to sense and respond to metabolic changes and make subsequent decisions about healthspan and lifespan extension.

### 3.1 Overview of Mitochondrial Function and Biological Aging

Mitochondria are dynamic double-membrane organelles present in most eukaryotic cells and are often regarded as the powerhouses of these cells. They regulate essential cellular metabolic processes, including ATP production, calcium homeostasis, ROS production and scavenging, apoptosis, mitophagy, and iron-sulfur cluster biogenesis. Over decades, good evidence has emerged linking mitochondrial function breakdown to biological aging. In 1980, the review by Miquel et al. offered the first insight into mitochondrial senescence and its possible relation to aging-related changes in other cellular organelles (Miquel et al., 1980). Furthermore, in 1989, Linnane et al. work led to the proposal that the cytosolic accumulation of mitochondrial DNA mutations increases with an advance in age and is a significant contributor to biological aging and age-related degenerative diseases (Linnane et al., 1989). These noble observations, including those of others (Papa, 1996), led to the postulation of the mitochondrial theory of aging, which states, “free radical species generated within the mitochondria promote the accumulation of genetic alterations of mitochondrial DNA (somatic mutations), culminating in defects in mitochondrial DNA encoded proteins, followed by a breakdown in electron transfer activity and oxidative phosphorylation” (Wallace, 2010; Schon et al., 2012; Bratic and Larsson, 2013). This theory reaffirms that age progression declines mitochondrial function and is accompanied by alterations in mitochondrial morphology, content, and oxidative phosphorylation capacity. From this view, the scientific community has universally agreed that mitochondrial dysfunction is a hallmark of biological aging and that interventional development strategies that directly or indirectly manipulate mitochondrial functions and morphology can positively prevent biological aging. However, it is worth mentioning that the mitochondrial free radical theory has been heavily questioned and opposed in recent times [reviewed in ref. (Bratic and Larsson, 2013)]. Even so, evidence still exists that, for example, some mitochondrial DNA mutations caused by random errors during mitochondrial DNA replication contribute to accelerated cellular aging. Also, errors caused by repairing damaged mitochondrial DNA bases are often detected in aging humans and other organismal species (Kauppila et al., 2018). A significant consequence of mitochondrial DNA defects in man is neuronal energy failure, leading to encephalopathy, neurodegenerative diseases, and premature neural aging. Defective mitochondrial DNA can also have a variety of other systemic manifestations, such as cardiac myopathy and diabetes. More recently, research has connected aging phenotypes such as reduced fertility, hair greying and loss, and stem cell dysfunction to aberrant mitochondrial biogenesis caused by impaired retrograde signaling mediated by dependent and independent mitochondrial metabolic genes and factors (Ryan and Hoogenraad, 2007). This evidence still confirms the pivotal role of mitochondrial dysfunction in biological aging and age-related diseases. Below, we briefly set out the connection between the arms of mitochondrial function and accelerated biological aging.

#### 3.1.1 Mitochondrial Biogenesis

A mitochondrion is an organelle with its genome. Mammalian mitochondrial DNA is a circular double-stranded and intronless molecule of ∼16.5 kb in multiple copies per cell. It is usually packaged into DNA-protein structures known as mitochondrial nucleoids and is generally responsible for gene expression needed to maintain mitochondrial functional integrity (Rahman and Rahman, 2018). For instance, within the mitochondrial DNA sequence, 37 genes encode 13 protein subunits essential for the oxidative phosphorylation system. Mitochondrial biogenesis, defined as the growth and division of pre-existing mitochondria to make new mitochondria, is a highly coordinated process dependent on an ordinated balance in mitochondrial fusion and fission processes, normal replication of mitochondrial DNA, and several transcriptional proteins encoded by the nuclear genome (Bhatti et al., 2017). Today, good evidence pinpoints the peroxisome proliferator-activated receptor-gamma coactivator 1 alpha (PGC-1α) as the central regulator of mitochondrial biogenesis. Activation of PGC-1α modulates the nuclear-encoded protein levels of multiple mitochondrial DNA replication, transcription, and packaging factors (DNA polymerase gamma, mitochondrial single-stranded binding protein, twinkle helicase, and mitochondrial transcription factor A). In addition to the above mitochondrial biogenetic function of PGC-1α, in mammals, PGC-1α regulates cellular metabolism, and its physiological level is influenced by nutrient availability. Evidently, some metabolic regulators, such as mTOR, SIRT1, and AMPK, can stimulate PGC-1α and its dependent downstream signaling activation. In aging research, mitochondrial biogenesis is impaired because regulators of mitochondrial biogenesis levels are drastically reduced at transcriptional and posttranscriptional levels with aging. Also, mitochondrial biogenesis impairment correlates with the progression of multiple age-dependent diseases such as neurological disorders, cardiovascular and metabolic disorders, cancer, and premature aging disorders (López-Lluch et al., 2008; Yuan et al., 2016).

#### 3.1.2 Mitochondrial DNA Copy Number and Mutation

The mitochondrial DNA, as described briefly in an earlier subtopic, is necessary for the normal functioning of mitochondria. In view of this, inherited or acquired mutations trigger various mitochondrial-dependent disorders, including mitochondrial diseases. Numerous human clinical and rodent experimental studies suggest somatic mitochondrial DNA mutations as a causal factor for the acceleration of biological aging. Also, mitochondrial DNA mutation levels show a good correlation with aging phenotypes (Bagge et al., 2020). In rodents, for instance, deficiency in DNA polymerase gamma caused a progressive amassing of mitochondrial DNA mutations, which correlated with apoptotic markers and untimely accelerated aging syndrome with decreased lifespan and fertility (Kujoth et al., 2005). Moreover, mitochondrial DNA copy number, defined as a measure of mitochondrial genomes per cell, has emerged as an important marker for determining mitochondrial function. Currently, studies of aging humans and animal models have associated mitochondrial DNA copy number with several aging-related diseases. Because of this, it is being considered that the absolute mitochondrial DNA copy number level is a critical factor in human pathology and age (Mengel-From et al., 2014; Ding et al., 2015; Knez et al., 2016; Zhang et al., 2017). Poor health in the elderly, including a decrease in cognitive and physical activity, was associated with a high mortality rate and low mitochondrial DNA copy number in peripheral blood (Mengel-From et al., 2014). However, particularly on mitochondrial DNA copy number, multiple studies have also detected or observed no apparent correlation between mitochondrial DNA copy number and aging or aging-related diseases (Miller et al., 2003; Frahm et al., 2005; He et al., 2014; van Leeuwen et al., 2014; Wachsmuth et al., 2016). Thus, the current understanding of the subject matter does not strongly clarify whether low mitochondrial DNA copy number is associated with an adverse or advantageous impact on lifespan and aging-associated diseases. Therefore, further experiments are required to confirm that low mitochondrial DNA copy number is linked to the aging process.

#### 3.1.3 Mitochondrial Fission

Is a divisive process in which mitochondrial DNA replicates to produce new daughter mitochondria with inherited parental mitochondrial phenotypes. This process is regulated by intrinsic mitochondrial proteins that function as anchors and extrinsic proteins, from the cytosol, as initializers. The critical protein at the center of the mitochondrial fission mediation process is the dynamin-related protein 1 (Drp-1). However, other binding and collaborative-protein partners of Drp-1, such as mitochondrial fission factor, mitochondrial dynamics protein−49 and−51, fission protein-1 (FIS1), etc., contribute significantly to the initiation and progression of the fission process (Losón et al., 2013; Atkins et al., 2016). On the grounds of combating aging’s detrimental effects, mitochondrial fission is claimed to be incredibly important later in life. Deregulation or reduction of these mitochondrial fission proteins is believed to occur with age. Additionally, their reduced levels adversely impact aged people’s mitochondrial function and morphological quality. In line with this idea, studies have strongly reaffirmed that mitochondrial fission deteriorates with age. Moreover, the reactivation of fission-regulated protein expression improves lifespan by maintaining mitochondrial function and structural integrity (D’Amico et al., 2019; Byrne et al., 2019). For instance, the abrogation or deletion of Drp-1 reduces growth and causes atrophy, degeneration, and finally, death in animals (Favaro et al., 2019). In aged individuals, the loss of muscle mass is linked to the aging-related disease sarcopenia. Therapeutic interventions targeting mitochondrial fission dynamics by increasing the expression of Drp-1 were found to preserve and enhance muscle mass and strength (Standley et al., 19852017). A study has also shown that promoting Drp-1-mediated mitochondrial fission in midlife prolongs flies’ healthy lifespan (Rana et al., 2017). On the contrary, some studies have reported that reduced mitochondrial fission extends lifespan (Scheckhuber et al., 2007). Mitochondrial fragmentation contributes to mitochondrial dysfunction by promoting skeletal muscle insulin resistance, leading to aging-associated metabolic diseases such as metabolic syndrome and type II diabetes (Jheng et al., 2012). Drp-1 inactivation consistently enhanced the effect of decreased IIS to improve longevity in flies (Yang et al., 2011). Another study recently added that upregulation of mitochondrial fragmentation and Drp-1 levels promotes neurotoxicity, which is implicated in aging-neurodegenerative disorders such as Parkinson’s disease (Zheng et al., 2020). The highlighted studies show that promoting and reducing mitochondrial fission and its-related proteins arguably breaks down mitochondrial function and morphology integrity, contributing to accelerated aging. Perhaps the differences in the studies’ findings can be partly related to the study subjects (tissue or organism) and the study design employed. Regardless of the inconsistencies in findings, it is still evident that the understanding of mitochondrial fission in aging is still rudimentary and born on associative data with less robust causal-investigative mechanistic studies. Therefore, this warrants future studies on how environmental and genetic factors (known critical players in aging processes) alter mitochondrial fission-related proteins and how fission proteins reprogram to influence intrinsic mitochondrial-dependent and independent pathways to expand or decline aging-associated molecular events.

#### 3.1.4 Mitochondrial Fusion

Is a highly coordinated process mediated by dynamin-related GTPases located in the inner and outer mitochondrial membranes (Anton et al., 2019; Schuster et al., 2020). The inner mitochondrial membrane fusion is mediated by the specialized protein named optic atrophy 1 (OPA1), while mitofusin 1 (MFN1) and 2 (MFN2) mediate outer-mitochondrial membrane fusion. OPA1 inhibition or MFN1/2 knockout reduces mitochondrial fusion and promotes hyper-mitochondrial fragmented networks (Meyer et al., 2017). In cells, mitochondrial fusion is a conserved complementation process, essential to the prevention of mitochondrial stress by linking defective mitochondrial content with healthy mitochondria. This process enhances metabolic efficiency and metabolite exchange and promotes ATP production, activities partly mediated by AMPK and dietary restriction (Misko et al., 2010; Weir et al., 2017; Yao et al., 2019). Generally, during aging, the promotion of the expression of fusion-mediated regulatory proteins extends longevity by reducing oxidative stress and increasing metabolic fitness (Jacobi et al., 2015). For example, in flies, increased mitochondrial fusion extended the survival span of the adult flies through the activation of IIS and SCF^LIN−23^-modulated pathways (Chaudhari and Kipreos, 2017). Likewise, Kornicka et al. demonstrated that interventions promoting mitochondrial fusion, but not fission, are vital for reversing senescence and aging cells through decreased apoptosis and ROS accumulation (Kornicka et al., 2019). Furthermore, the enhancement of mitochondrial fusion in larvae and adult flies mitigated proteasome dysfunction-induced developmental lethality by stabilizing the proteome to prevent altered metabolic pathways and disrupted mitochondrial functionality. This further affirms that increasing age downregulates the ubiquitin-proteasome pathway to promote proteotoxic stress, deregulating cellular functionalities such as imbalanced mitochondrial dynamics, which drive most age-related diseases (Gumeni et al., 2019; Tsakiri et al., 2019).

#### 3.1.5 Mitochondrial Bioenergetics

Involves enzymatic and metabolic events regulating biochemical pathways of energy generation and transformation. During glycolysis, pyruvate is produced and oxidized to acetyl-coenzyme A, fueling the Krebs cycle, and subsequently powering up cellular respiration *via* reducing agents, NADH and FADH_2_, production. NAD^+^ and FAD^+^, derived from the oxidation of NADH and FADH_2_, respectively, are utilized as substrates by the mitochondrial electron transport chain to generate ATP *via* oxidative phosphorylation. The synthesized ATP molecules are exported to the cytosol, where they function to fuel diverse critical cellular functions and processes. Interestingly, electron movement within the mitochondrial oxidative phosphorylation system is driven by redox potential and proton gradient. Furthermore, oxygen consumption proceeds with inescapable ROS as a byproduct (van der Bliek et al., 2017). The link between mitochondrial bioenergetics and biological aging arose from disruptions in mitochondrial respiration, which resulted in erratic ROS generation, which disrupted the redox system balance, contributing to damage to organismal tissues and organs. However, despite the free-radical theory of biological aging being extensively debated, many free radicals, including ROS, still linger as contributory factors to aging-equated diseases, including cardiovascular and metabolic diseases, neuropathologies, etc., (Brand et al., 2013; Ochoa et al., 2018; Madreiter-Sokolowski et al., 2020). Besides, during upregulation of ROS, a decline in energy has been observed in the elderly, and it has been suggested that insufficiency of mitochondrial respiration accounts for this. Thus, optimal restoration of mitochondrial bioenergetics offers an avenue for managing numerous age-related diseases by improving ATP-dependent cellular functions and processes (Szeto, 2014).

#### 3.1.6 Mitochondrial Calcium Homeostasis

Regulation of calcium is an important cellular event, chiefly mediated by mitochondria in coordination with the endoplasmic reticulum. In the mitochondrion, mitochondrial-specific ion channels, for example, mitochondrial calcium uniporter and mitochondrial membrane potential, facilitate calcium entry into the mitochondrial matrix. Mitochondrial calcium homeostasis and signaling are essential for mitochondrial biology and other molecular and cellular functions in mammalian cells (Pathak and Trebak, 2018). However, calcium ions, either at lower or above threshold levels in the mitochondrial matrix, influence mitochondrial metabolic fate. Likewise, dysfunctional mitochondria potentiate mitochondrial calcium signaling (Salazar-Ramírez et al., 2020). During mitochondrial calcium overload, profound generation of ROS, increased mitochondrial apoptosis, contraction, and immoderation of mitochondrial membrane potential are observed. Correspondingly, studies have shown that undue mitochondrial calcium influx participates in pathophysiological conditions fundamental to several aging-dependent diseases (Jung et al., 2020). As such, mitochondrial and cytosol calcium overload in aged tissues and organs aggravates multiple aged-correlated diseases, and modulating calcium homeostasis and signaling to mitochondria have materialized as promising therapeutic strategies for promoting longevity (Burkewitz et al., 2020).

### 3.2 Overview of Nutrient-Sensing Pathways and Biological Aging

Nutrient-sensing pathways are currently the most influential pathways in aging biology research because of their broad scope and link to cellular metabolism. These nutrient-sensing pathways detect and respond to nutrient levels and initiate downstream cascade responses such as growth, energy, and reproduction. Currently, perturbing nutrient-sensing node pathways by caloric restriction (a reduction of caloric intake without apparent signs of malnutrition), pharmaceutical, dietary, or genetic intervention is one of the robust ways to slow aging processes to extend the human lifespan and protect against aging-related diseases. This has been demonstrated in several laboratory-adapted species like flies, yeast, mice, worms, and even in humans (Madeo et al., 2019). The most widely studied nutrient-sensing signaling pathways in relation to aging biology are SIRT/NAD^+^, AMPK, mTOR, and IIS pathways. Thus, we concisely discuss these nutrient-sensing metabolic pathways and evidence that they modulate the aging process below.

#### 3.2.1 Insulin/Insulin-Like Growth Factor 1 Signaling

Controls information about nutrient status to influence metabolism and growth. In 1993, a mutation within the DAF-2 gene, a homolog of the insulin-like growth factor 1 receptor, in *C. elegans* increased the lifespan twice as long as wild-type worms (Kenyon et al., 1993). This earlier observation sparked various research into the components of this pathway and their association with aging. Currently, multiple studies have reaffirmed the earlier observation by showing that suppression or mutations of components (insulin, growth hormone, insulin-like growth factor 1 receptor, downstream kinases, receptors, etc.) of the pathway expand lifespan in diverse model organisms (Kenyon, 2010; Sasako and Ueki, 2016; Blüher et al., 2003). Similarly, in humans, insulin-like growth factor 1 receptor variants and decreased insulin-like growth factor 1 receptor levels correlated with exceptional lifespan extension and quality of life (Martins et al., 2016; Flachsbart et al., 2017). Also, IIS activation can remodel mitochondrial network integrity and metabolic function, influencing diverse cells’ responses to insulin, thus triggering aging-related metabolic diseases such as insulin resistance and type 2 diabetes. Moreover, genetic mutations of growth hormone receptors have been identified and linked to insulin-like growth factor 1 signaling pathway reduction, stunted growth, and other aging-associated diseases (Guevara-Aguirre et al., 2011; Villela et al., 2020). However, despite the substantive evidence of the correlation of low insulin-like growth factor 1 components to accelerated aging, many studies have also reported otherwise in humans (Paolisso et al., 1997; Arai et al., 2008). Overall, the IIS nutrient signaling pathway is essential in aging, but the relational causal effects remain controversial. Thus, further robust studies on the characterization of tissue-specific functions of the IIS pathway will help us understand how best to target or modulate the pathway for longevity and healthy aging.

#### 3.2.2 Activated Protein Kinase

Is a serine/threonine kinase stimulated by energy stress (low ATP: ADP ratio) and two upstream regulators (liver kinase B1 and calcium/calmodulin-dependent protein kinase) (Carling, 2017; Oduro et al., 2020). Activated AMPK directs metabolism towards catabolism through varied stimulation of multiple pathways, including mTOR complex 1 signaling and internal homeostasis processes, such as lipid and mitochondrial homeostasis, through acetyl coenzyme A carboxylase and PGC-1α, respectively. Due to the profound role of AMPK in cellular metabolism (glucose, mitochondrial, and lipid metabolism), and its contribution to the maintenance of intracellular quality control processes (proteostasis, autophagy/mitophagy, apoptosis), dysregulation of AMPK is the prevalent causal factor for most cardio-metabolic disorders, particularly type 2 diabetes, atherosclerosis, cancer, etc., Interestingly, these cardio-metabolic disorders are age-dependent diseases, with prevalence and incidence rates increasing with advancement in age. Therefore, stimulating AMPK is a preferred therapeutic target, notably for anti-aging compounds (Steinberg and Kemp, 2009; Carling, 2017). Consistently, lifespan extension driven by AMPK is mediated by cAMP response element-binding protein, CREB transcription coactivator 1, energy levels, and insulin-like signals (Apfeld et al., 2004; Mair et al., 2011). Metformin is a popular type 2 anti-diabetic drug whose unique therapeutic effects have been aligned with the activation of AMPK. In relation to this, type 2 diabetic patients treated with metformin had a median lifespan of 15% longer than the matched control group (Steinberg and Kemp, 2009; Carling, 2017), further extending the evidence base for AMPK as an anti-aging nutrient molecule.

#### 3.2.3 Sirtuins

Are primarily mammalian protein deacetylases. Usually, sirtuins utilize nicotinamide adenine dinucleotide (NAD^+^) as a coenzyme to pull out acyl groups from numerous proteins. To date, seven sirtuins (SIRT1-7) are known to be present in humans and mice. SIRT1/2 and SIRT6/7 are deacetylases, while SIRT3, 4, and 5 are usually involved in fatty acid oxidation in mitochondria. Recently, sirtuins have gained the necessary attention because it is generally claimed that overexpression of some specific sirtuins or their orthologues increases lifespan in yeast, worms, flies, and humans (Imai and Guarente, 2014; Wątroba et al., 2017). SIRT1 is the most widely and extensively studied sirtuin in aging research. It is customarily postulated that SIRT1 expands lifespan and quality of life chiefly through the deacetylation and stimulation of PGC-1α, culminating in the modulation of mitochondrial biogenesis, cell cycle, apoptosis, autophagy, lipid metabolism, and endothelium-dependent vasodilation (Imai and Guarente, 2014; Bonkowski and Sinclair, 2016). Given this, SIRT1 is regarded as a promising intervention target to restore metabolic dysregulation to prevent aging-related metabolic disorders such as type 2 diabetes, metabolic syndrome, atherosclerosis, and heart diseases (Lee et al., 2009; Liang et al., 2009). Besides, NAD+, the coenzyme used by sirtuins, has a broad impact on cellular physiological processes. Changes in its levels eventually have an impact on biological events. Age-dependent reduction of cellular NAD+ is observed in humans and other organismal species. This is attributed to the inequality between NAD+ production and consumption as age increases. Moreover, studies have revealed that reduced NAD+ amounts correlated with multiple aging-related diseases, and precursor supplementation exerts profound beneficial effects against aging-related diseases and promotes longevity (Katsyuba and Auwerx, 2017; Yaku et al., 2018).

#### 3.2.4 Mammalian Target of Rapamycin

Are well recognized as master regulators of growth and survival. mTOR complexes are under intensive research in aging (Saxton and Sabatini, 2017). mTOR complexes comprise mTOR complex 1 and mTOR complex 2. Both complexes contain mTOR and mLST8/Deptor. mTOR complex 1 has additional components such as Raptor and PRAS40, and mTOR complex 2 contains Rictor, mSIN1, and Protor. mTOR complex 1 activation stimulates anabolic processes, lipid biosynthesis, and protein synthesis *via* mTOR complex 1-mediated phosphorylation of its downstream substrates ribosomal protein S6 kinase one and eukaryotic translation initiation factor 4E binding protein 1. Moreover, mTOR complex activation suppresses lipolysis and autophagy (the degradative signals often likened to cellular homeostasis maintenance) (Johnson et al., 2013; Saxton and Sabatini, 2017). Currently, studies have demonstrated that over-stimulation of mTOR complex 1 signaling drives multiple aging-associated diseases, particularly late-onset diabetes type 2 (Selman et al., 2009; Zoncu et al., 2011; Mossmann et al., 2018). On the other hand, mTOR complex 2 regulates cell survival and cytoskeleton restructuring of actin filaments and glucose and lipid metabolism *via* protein kinase B-independent and dependent mechanisms. Its suppression reduces lifespan and correlates with metabolic changes, negatively impacting longevity (Lamming et al., 2012). Moreover, one of the challenges that have been observed with the development of mTOR complex 1 inhibitors in anti-aging therapies is the chronic non-specific suppression activity towards mTOR complex 2 (Sarbassov et al., 2006). This shows that effective treatment strategies against aging-related diseases and improving lifespan might rely on developing specific inhibitors that selectively arrest mTOR complex 1 signaling outputs. Furthermore, despite the individual roles of mTOR complexes, a growing body of literature shows that mTOR participates in regulating pillars of aging (loss of proteostasis, mitochondrial dysfunction, cellular senescence, and stem cell exhaustion and function) [(review in ref (Papadopoli et al., 2019)].

### 3.3 Dietary and Natural Products Target Mitochondrial Dysfunction and Nutrient-Sensing Metabolic Pathway

**FIGURE 3:**
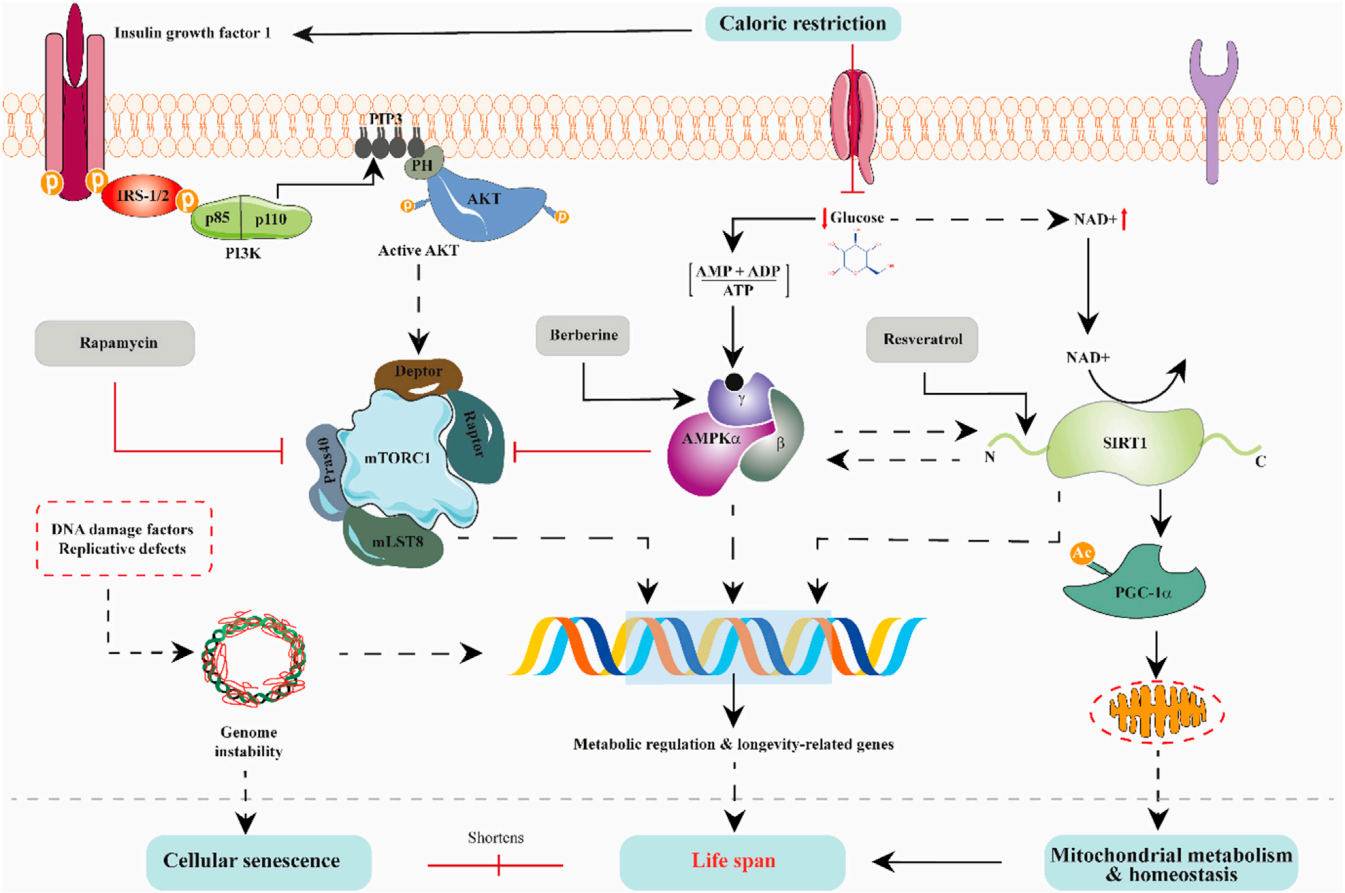
The interlinked regulation of nutrient-sensing pathways. The IGF1, mTORC1, AMPK, and SIRT1 signaling pathways modulate aging *via* interacting with one another. Nutrient availability exerts diverse interrelated influences on these pathways. For instance, decreased intracellular glucose stimulates AMPK and promotes NAD + levels, culminating in inhibition of mTORC1 activity and enhancement of SIRT1 activity. Suppression of mTORC1 activity is projected to be beneficial in extending life because inhibition tilts the balance towards mTORC2, enhancing critical age-dependent proteins needed for metabolic regulation. Also, IGF1 signaling activation is known to lower lifespan through mTORC1; however, under caloric restriction conditions, AMPK inhibits mTORC1. Thus, extending life. On the other hand, SIRT1 activation, directly by AMPK or indirectly by NAD + under nutrient restriction, promotes mitochondrial metabolism and homeostasis *via* PGC1*α*. Stabilization of mitochondrial metabolism and homeostasis is known to prolong lifespan. Conversely, in cells, DNA instability resulting from replicative defects and damage to DNA can disorient transcription of metabolic and longevity-related genes and trigger cellular senescence, thus shortening lifespan. In natural product therapeutic pharmacology, rapamycin inhibits mTORC1 activity while berberine and resveratrol activate AMPK and SIRT1 activity, respectively.

The above-discussed body of works demonstrates that alterations in organisms’ mitochondrial and nutrient-sensing metabolic pathways or processes can lead to aging acceleration. Strikingly, most dietary, and natural product interventions purported to increase healthspan and, to some extent, lifespan act on mitochondrial metabolic processes and nutrient-sensing longevity mediators. For example, natural dietary drugs such as rapamycin, resveratrol, berberine, curcumin, and many more have ant-aging effects and are frequently used in laboratory experiments as mechanistic tools to expound molecular pathways underpinning aging. In point of fact, rapamycin (a natural product, first isolated from *Streptomyces hygroscopicus*) is an mTOR complex 1 inhibitor that extends life in worms, flies, and mice (Fischer et al., 2015; Kennedy and Lamming, 2016; Schinaman et al., 2019). Rapamycin is often used on laboratory-adapted metabolic tissues to prove that modulation of mTOR complex 1 and its closely associated nutrient-sensing signaling pathways are appropriate to improve metabolism and lifespan (Kennedy and Lamming, 2016). In addition to rapamycin, berberine and resveratrol are AMPK and SIRT1 modulators, respectively, typically used to describe AMPK/SIRT1/PGC1α energy-sensing pathway regulatory effects on mitochondrial biogenesis and other mitochondria-dependent functions (Figure 3). Taken together, as summarized in Table 1, bioactive dietary and natural products have strong links with aging hallmarks, mitochondrial dysfunction, and deregulated nutrient-sensing networks. They act tissue-autonomously and tissue-non-autonomously to regulate metabolic communications of mitochondria and nutrient-sensing networks to modulate organismal longevity. However, additional translational evidence with a robust study design on tolerability, safety, effectiveness, and off-target effects in the aging population would enhance the clinical potential of dietary and natural products in anti-aging-related treatment schemes.

**TABLE 1:** Natural products and/or dietary supplements target mitochondria and metabolic mediators and pathways.

Natural product category	Name of natural product	Regulatory effects on mitochondria	Modulation effects on nutrient-sensing and cellular trash pathway	References
Anthraquinone	Purpurin	↓ROS, mitochondrial membrane potential, and abundant ATP production	↑AMPK	Nam et al. (2019)
Benzylisoquinoline alkaloid	Berberine	Protects mitochondrial structure and function	↑SIRT1/3, AMPK	Sun et al. (2018), Javadipour et al. (2019), Rong et al. (2021)
		↓Mitochondrial ROS levels and activity of complex I		
		↑Activity of complexes II, IV, and mitochondrial membrane potential		
		↓ATP abundance and inhibits apoptosis		
Carotenoid alcohol	Zeaxanthin	↑Mitochondrial DNA content and mitochondrial biogenesis genes	↑AMPK	Liu et al. (2019)
		↓Mitochondrial oxidative damage, mitochondrial superoxide ions, and intracellular ROS.		
		↑Mitochondrial membrane potential		
Diarylheptanoid	Curcumin	↑Mitochondrial DNA copy number, MFN1/2, OPA1, and ATP production	↑SIRT1 activity, AMPK phosphorylation	Kuo et al. (2012), Reddy et al. (2016), Mañas-García et al. (2020)
		Restored mitochondrial oxidative metabolism		
		↓Drp1 and FIS1		
		↑PGC-1α and TFAM levels		
Flavanol	Epigallocatechin-3- gallate	↑Mitochondrial DNA replication	↑AMPK, SIRT1	Liu et al. (2015), Lee et al. (2017), Hsieh et al. (2021)
		Restored mitochondrial DNA copy number		
		↑Mitochondrial biogenesis	↓mTOR	
		↑PGC-1α and TFAM levels		
Flavanonol	Dihydromyricetin	↑ATP content, mitochondrial DNA content, and citrate synthase activity	↑SIRT3	Wei et al. (2019); Li et al. (2022)
		↓Mitochondrial MnSOD activity, ROS level, and caspase-3 activity	Activates AMPK/mTOR pathway	
Flavone	Baicalin	Stabilizes the mitochondrial membrane	Activates SIRT1/AMPK/mTOR pathway	Chan et al. (2016), Deng et al. (2020), Chen et al. (2021b)
		↓Discharge of cytochrome c from mitochondria	↑AMPK	
		Activates mitochondrial autophagy		
		Induces mitochondrial fission and mitochondrial impairment		
Flavone	Luteolin	↑Mitochondrial membrane potential, ATP content, citrate synthase activity, and complexes I/II/III/IV/V activities	↑SIRT1	Yang et al. (2013), Hu et al. (2016), Sayed et al. (2020), Wu et al. (2020)
		↑Drp1-dependent mitochondrial fission	↓Insulin-like growth factor signaling. Activate AMPK/mTOR autophagy pathway	
Flavonol	Quercetin	↑Mitochondrial membrane potential, oxygen consumption, ATP, mitochondrial copy number, and mitochondrial bioenergetics capacity	↑AMPK, SIRT1	Ay et al. (2017), Li et al. (2018), Qiu et al. (2018)
		Restores mitochondrial cytochrome c, malondialdehyde, and superoxide dismutase levels	Activates Akt/mTOR/p70S6K autophagy pathway	
		↑PGC-1α levels		
Flavonol glycoside	Icariin	↑Mitochondrial motility, index and mitochondrial length and size, mitochondrial enzyme pyruvate dehydrogenase-E1α, MFN2, and mitochondrial transport	↑SIRT1, AMPK	Zhu et al. (2010), Chen et al. (2016)
		↓Mitochondrial fission protein Drp1 and mitochondrial fragmentation	Stimulates AMPK/mTOR autophagy pathway	
Isoflavone	Daidzein	↑Mitochondrial genes such as COX1 and CYTB and mitochondria content	↑SIRT1, AMPK	Keung et al. (1997), Yoshino et al. (2015)
		↓Mitochondrial aldehyde dehydrogenase	Stimulates mTOR activity	
		↑PGC-1α and TFAM levels		
Isoflavone glycoside	Puerarin	↑Mitochondrial density, tricarboxylic acid cycle, and oxidative phosphorylation function	↑SIRT1	Chen et al. (2018), Peng and Liu (2022)
		↑PGC-1α levels	Stimulates AMPK/SIRT1 pathway	
Keto-carotenoid	Astaxanthin	Maintains mitochondrial tubular structure function and normalizes mitochondrial membrane potential	Modulates the insulin signaling pathway by targeting DAF-16	Yu et al. (2019), Liu et al. (2021)
		↓Mitochondrial fragmentation and depolarization, and apoptotic death	Acts as a dietary restriction mimic	
		↑PGC-1α and TFAM levels		
O-methylated flavonol	Isorhamnetin	↑Mitochondrial DNA/nuclear DNA ratio and mitochondrial DNA replication	↑AMPK, SIRT1	Lee and Kim, (2018)
		↑PGC-1α and TFAM levels		
Phenolic Glycoside	Salidroside	↑Mitochondrial DNA copy and electron transport chain proteins	↑SIRT1, AMPK, SIRT3	Xue et al. (2019), Shan et al. (2022)
		↑PGC-1α	Activates PI3K/Akt/mTOR autophagy pathway	
		↑Mitochondrial membrane potential		
		Inhibits mitochondrial cytochrome c release		
		Activates mitochondrial biogenesis		
Polymethoxylated flavone	Nobiletin	↑MFN2, OPA1, and partial promote mitochondrial depolarization	↑SIRT1, AMPK	Lee et al. (2018), Dusabimana et al. (2019)
		↓Drp1, Mitochondrial calcium overload, and ROS generation	Activate AMPK autophagy pathway	
Polyphenol-biflavonoid	Theaflavin	↑Mitochondrial DNA copy number	↑SIRT1	Tong et al. (2018), Qu et al. (2021)
		↓PGC-1 *β* mRNA and PRC levels	Activates CaMKK2-AMPK signaling	
		↑Mitochondrial biosynthesis and abundance		
Polyphenolic	Trans-δ-viniferin	Preserve mitochondrial membrane potential	↑SIRT1	Zhao et al. (2016)
Simple Phenol	Hydroxytyrosol	↑Mitochondrial respiratory chain complexes I/II/IV express and the activity of complex I	↑AMPK, SIRT1	Zheng et al. (2015)
		↑Mitochondrial energetics and biogenesis		
Stilbenoid	Pterostilbene	↑Mitochondrial respiratory chain complexes III and V and mitochondrial membrane potential	↑AMPK	Zhang et al. (2012), Kosuru et al. (2018)
		↓Mitochondrial cytochrome c release		
Triterpenoid saponin	Ginsenoside Rg1	↑Mitochondrial length, activity, and MFN2 expression	↑AMPK	Dong et al. (2016), Lee et al. (2019)
		↓Fragmented mitochondria		

↑, Increase/Promote; ↓, Decrease/Reduce; AMPK, AMP-activated protein kinase; ATP, adenosine triphosphate; COX1, Cytochrome c oxidase 1; Drp1, Dynamin-related protein-1; FIS1, Mitochondrial fission one protein; MFN1/2, Mitofusin1/2; MnSOD, manganese superoxide dismutase; NA, Non-applicable/Not reported; NRF1, Nuclear respiratory factor 1; OPA1, Optic atrophy 1; PGC-1α, Peroxisome proliferator-activated receptor-gamma coactivator-1alpha; PRC, PGC-related coactivator; ROS, reactive oxygen species; SIRT1/3, Sirtuin1/3; TFAM, Mitochondrial transcription factor A.

## 4 Clinical Utility and Control of Biological Aging and Age-Related Diseases by Dietary Supplements and Natural Products

Dietary supplements are products not sold as conventional foods but are meant to complete or enhance a diet containing one or more vitamins, minerals, amino acids, or botanicals (except tobacco). On the other hand, natural products, which for commercial purposes are usually somewhat referred to as dietary supplement products produced from natural sources, are naturally derived chemical compounds that can be chemically complex with naturally varying compositions. Over the years, the dietary supplement and natural products market has seen remarkable growth and claims of conferring health benefits on humans have been under intensive scrutiny by local, national, and international organizations and research institutions. Although various governmental regulatory authorities and research institutions support dietary supplements and natural products with enough evidence on potential health benefits, claims such as diagnosing, mitigating, treating, curing, or preventing disease by natural products and dietary supplements are usually not welcome (Kuszak et al., 2016).

In the current era of modern science, dietary supplements and natural products have seen remarkable scientific and technological innovations in assessing their effectiveness, safety, and efficacy in various aging health outcomes, such as memory loss, menopause, cognitive decline, and skin aging. For instance, clinical trials involving minerals, lutein, vitamins, zeaxanthin, and beta-carotene supplementation show they can safely and effectively slow accelerated aging-related cataracts and macular degeneration progression (Age-Related Eye Disease Study Research Group, 1999; Chew et al., 2012). Furthermore, the rising use of nutritional and natural products among the elderly suggests dietary and natural products as alternative and complementary treatment options needed to support healthy aging and control aging’s unwanted effects. Besides, various natural compounds are now being employed in the laboratory to pave the way for unraveling the evolutionarily conserved biological processes of aging. Interestingly, most natural products show promise as epigenetic modifiers, senolytics, and senostatics in cellular aging (Evans et al., 2020b; Ikonne et al., 2020; Kaur et al., 2020).

### 4.1 Control of Biological Aging and Aging-Dependent Diseases by Dietary Supplements

#### 4.1.1 Vitamins

Are organic molecules considered essential micronutrients because most organisms cannot synthesize them entirely or in sufficient quantities, and they must be obtained in diets in lesser amounts for the proper functioning of an organism’s metabolism. Interest in the therapeutic potential of vitamin supplementation, particularly vitamin D, for human longevity and to reduce the risk of aging-related abnormalities and all-cause mortality has been a hot topic in clinical studies (Garay, 2021). This interest stems from basic laboratory studies showing that vitamin D and its metabolites postpone age-related diseases by inhibiting oxidative stress, supporting innate immune responses, inhibiting DNA damage and inducing DNA repair mechanisms, regulating mitochondrial and glucose metabolism, suppressing cellular senescence, increasing telomerase activity, etc., (Bima et al., 2021; He et al., 2021; Yang et al., 2022). These notable findings from laboratory studies suggest that vitamin D consumption has anti-aging properties. Moreover, low circulating 25-hydroxyvitamin D levels (a vitamin D status biomarker) have been linked to accelerated aging, cognitive impairment, or dementia in aging populations and an increased risk of age-related chronic illnesses and death (Conzade et al., 2017; Ju et al., 2018). In humans, the subject of vitamin D intake increasing lifespan remains inconclusive. Its role in preventing age-related chronic diseases has produced heterogeneous results, with some showing protective outcomes and others showing null findings (Pittas et al., 2019; Dhaliwal et al., 2020; Grübler et al., 2021). One likely explanation for these heterogeneous findings is the difference in study design. Another way to look at it is that some people are incredibly resilient to treatment, and these contradictory findings are attributable to individual differences in response to vitamin D treatment. Nonetheless, a recent systematic review and meta-analysis have also found that vitamin D intake significantly reduces the risk of acute respiratory infections (Jolliffe et al., 2021). Aside from vitamin D and its metabolites, other vitamins such as B, K vitamins, etc., have been studied as supplements that support healthy aging and enhances quality of life. However, the story of these vitamins’ intake in humans is like that of vitamin D (Rønn et al., 2016; Lees et al., 2021). Today, vitamins are now often strategically combined with herbs, minerals, and other natural products to maximize their benefits against accelerated biological aging. Vitamin B12, together with bacopa, lycopene, and astaxanthin, for example, effectively alleviated cognitive changes related to brain aging (Crosta et al., 2020). This shows that refining vitamin interventions by combining them with other health supplements might be good enough for clinical utilization.

#### 4.1.2 Minerals

Are essential to health and well-being and are usually obtained from diets. There are two kinds of minerals needed by the body for diverse reasons: macro-minerals and trace minerals. These minerals, especially calcium and zinc (here, the discussion is limited to these two mineral ions), have indispensable roles in ensuring the homeostatic balance of an organism. Because of their essential roles and possible impact on public health, several health institutions have set their tolerable values for daily, weekly, and monthly intakes in different age-population groups and countries (DeSalvo, 2016). Deficiencies in some mineral ions have been noted and linked to age-dependent diseases in aged individuals. Similarly, abnormal levels or signaling of some mineral ions in the elderly have equally been equated to the development and progression of age-related human diseases. Metabolic processes tightly regulate calcium homeostasis. However, for example, during accelerated aging, calcium levels fluctuate massively. That said, dysregulated calcium levels are linked to the acceleration of cellular aging. Also, calcium-related alterations during aging are considered a causative factor for neurological degenerative diseases. Parkinson’s disease, for example, is thought to be a manifestation of calcium-accelerated cellular aging. Thus, evidence suggests that calcium channel antagonist use is likely to slow Parkinson’s disease and promote healthy aging (Chen et al., 2021a). There is evidence that elderly individuals are at risk of calcium deficiency. On the other hand, adequate calcium dietary intake can reduce the risk of falls, osteoporosis, and fractures in the elderly population (Iuliano et al., 2021; Wallace et al., 2021). Like calcium, zinc is a mineral ion that supports healthy aging and promotes healthspan. Zinc has many biological effects and functions, including antioxidant, anti-inflammatory, immune modulation, DNA damage response, protein synthesis, apoptosis, etc., Nonetheless, zinc insufficiency, which presents as various physiological alterations in organs and cellular function, is often observed throughout accelerated aging, and contributes to human age-related disorders. This close association between zinc and age has made dietary zinc supplementation a therapeutic option for controlling age-related health conditions, including neurological disorders, infectious diseases, age-related macular degeneration, etc., (de Almeida Brasiel, 2020; Emri et al., 2020). In summary, a lot of animal literature proposes that zinc and calcium intake positively control accelerated cellular aging. However, in humans, it remains uncertain whether calcium and zinc supplementation are helpful or not in age-related health conditions, despite the prospects they have in slowing the aging process.

#### 4.1.3 Long-Chain Polyunsaturated Fatty Acids

Are fatty acids with 18 carbons or more and depending on where the initial double bond from the methyl end group of the fatty acid is positioned, it can be either omega-3 (ω3) or omega-6 (ω6) long-chain polyunsaturated fatty acids. The human body cannot produce these long-chain polyunsaturated fatty acids, so they must be obtained through diet. According to multiple clinical investigations, long-chain polyunsaturated fatty acids supplementation is thought to safeguard human health by influencing biological activities, notably aging processes, because intake of long-chain polyunsaturated fatty acids is associated with a decreased risk of age-related disorders (Wu et al., 2017; Arslan et al., 2019). The ω3 fatty acids docosahexaenoic and eicosapentaenoic are among the most researched long-chain polyunsaturated fatty acids in accelerated aging; thus, we limit our discussion to these ω3 fatty acids. Many aspects of aging studies have highlighted the health benefits and reduced risk of mortality associated with treatment with ω3 fatty acids across all individuals, particularly the elderly and laboratory-adapted aged species. Indeed, evidence suggests that higher circulating levels of ω3 fatty acids are associated with a lower risk of premature death from age-related diseases such as cardiovascular disease, cancer, and other causes (Harris et al., 2021). Moreover, several observational studies have linked dietary ω3 fatty acid consumption with cognitive function improvements or established a relationship between dietary ω3 fatty acid intake and cognitive performance in healthy and aging populations (Del Brutto et al., 2016). The mechanisms of action of ω3 fatty acids in aging are complex because of their diverse effects on multiple organs and cellular systems. That said, they have been shown to significantly lower age-related defects as well as have anti-inflammatory, antiapoptotic, antioxidant, and endothelial vasodilator actions (Kuszewski et al., 2017). In neurodegenerative disease models, ω3 fatty acids participate in neuron growth, memory formation, lipid raft organization, synaptic membrane function, myelination maintenance, photoreceptor biogenesis and function, and neuroinflammation reduction and neuroprotection (Joffre et al., 2019; Powell et al., 2021). While, in cardiovascular disease models, ω3 fatty acids participate in vasorelaxation, vascular endothelial function, lipid metabolism and thrombosis, and cardioprotection (Liao et al., 2021). Furthermore, supplementation with ω3 fatty acids has been linked to improvements in glucose and lipid disorders *via* the regulation of transcription factors peroxisome proliferator-activated receptorsα/δ. Also, ω3 fatty acids supplementation was additive in reducing hepatic steatosis when combined with caloric restriction (Kelley, 2016). Despite all the intensive research on the health benefits of ω3 fatty acids and their beneficial effects on influencing accelerated aging and aging-related functional declines, treatment efficacy associated with omega-3 fatty acids remains controversial due to inconsistent findings in humans. For example, a recent large-scale clinical study, that is the VITAL-DEP (Vitamin D and Omega-3 Trial-Depression Endpoint Prevention) randomized clinical trial, noted that treatment with ω3 fatty acids in adults [mean age, 67.5 (SD, 7.1) years] does not prevent depression but rather increases the risk of depression or clinically relevant depressive symptoms (Okereke et al., 2021). This concluding outcome made by the VITAL-DEP trial is similar to other previous clinical trials and studies that have reported null findings for ω3 fatty acid treatment in adults with or without any functional physiological decline and/or memory complaints (Andrieu et al., 2017; Danthiir et al., 2018; Rolland et al., 2019). On the other hand, several recent randomized controlled trials and prospective studies also prove that ω3 fatty acid supplementation improves cognition, depressive symptoms, mood, etc. (Tokuda et al., 2017; Jiang et al., 2018; Chang et al., 2020). These inconsistent findings presented by individual randomized clinical trials show that we still do not understand how ω3 fatty acids shape the aging environment in humans outside of basic laboratory studies. Therefore, we need to generate well-controlled clinical trials that will account for real-world parameters for ω3 fatty acid supplementation in humans, particularly in the aged population.

All in all, these data show that obtaining enough D, B, and K vitamins, calcium, zinc, and ω3 fatty acids not only has promising effects during aging—but also provides a shift in thinking that these supplements can complement modern medicines, which do not materially slow down aging processes to extend an individual’s lifetime and promote healthy aging. However, because of inconsistencies in human findings, large-scale, high-quality, and real-world clinical trials are required for more decisive discussion and to fully exploit the advantages of these supplements throughout aging.

### 4.2 Control of Biological Aging and Aging-Dependent Diseases by Natural Products

#### 4.2.1 Berberine

Is a well-studied natural product against several aging-related metabolic diseases. It is an isoquinoline quaternary alkaloid distributed in the roots, rhizomes, stems, and bark of several botanicals or herbs like *Coptis chinensis* (Chinese goldthread), *Coptis rhizome*, *Berberis vulgaris*, *Argemone mexicana*, etc., In aging research, berberine has been shown to extend the lives of several laboratory-adapted species. Regarding aging-related diseases, berberine is known to exert numerous beneficial outcomes in individuals with diabetes type II because of its profound effects on alleviating endothelial dysfunction and lipid metabolic imbalance and improving glycemic control, insulin sensitivity, and insulin secretion *via* targeting KCNH6 potassium channels (Zhang et al., 2008; Harrison et al., 2021; Zhao et al., 2021). Aside from diabetes type II, berberine has been demonstrated to have neuroprotective effects on aging-cognitive disorders and is also considered a potent chemosensitizer and chemoprotector for numerous cancers (Shang et al., 2020; Devarajan et al., 2021). Mechanistic insights into berberine activity in accelerated aging show that berberine suppresses the mitochondrial electron transport chain and premature cellular senescence to control the deleterious responses during aging. Berberine mediates its beneficial effects through AMPK activation, which sets downstream events in motion, including SIRT1 activation and mTOR suppression to trigger autophagy. This response promotes mitochondrial biogenesis to sustain mitochondrial capacity and improve mitochondrial-dependent metabolic processes, promoting healthy aging (Xu et al., 2017). Taken together, these studies demonstrate that berberine can slow aging by targeting nutrient metabolic signaling and preventing physiological declines of defined hallmarks of higher age.

#### 4.2.2 Luteolin

Is a natural flavone compound first derived from *Reseda luteola* but has also been isolated from *Salvia tomentosa*, and it is often distributed in leaves. Various dietary foods, such as broccoli, carrots, rosemary, and dandelion, contain some appreciable amounts of luteolin. Chronic inflammation and oxidative stress influence how quickly people age. Luteolin has emerged as a natural product that modulates oxidation and inflammation with beneficial outcomes in several age-dependent human diseases (Gendrisch et al., 2021). Moreover, data indicate that luteolin can effectively reduce photobiological aging by regulating the SIRT3/ROS/MAPK signaling axis (Mu et al., 2021). Luteolin is therapeutically valuable in several aging-related diseases, particularly Alzheimer’s disease and other cognitive disorders. These beneficial therapeutic outcomes of luteolin were associated with its neuroprotective and neuro-anti-inflammatory responses. Various biological clean-up mechanisms that combat accelerated biological aging and aging-related diseases have been put forward for luteolin at the cellular level. For example, luteolin was found to inhibit endoplasmic reticulum stress, IL1β production, and CD68 expression in microglia in the brain of an Alzheimer’s disease mouse model (Tana and Nakagawa, 2022). Also, luteolin is a SIRT6 agonist with modulation effects on Alzheimer’s disease, aging, diabetes, inflammation, and cancer (Akter et al., 2021). In addition to the above, using neuronal cell-based high-throughput for mitochondrial function enhancers, Naia L et al. discovered luteolin as a modulator of mitochondria-endoplasmic reticulum coupling, whose effects have the potential to revert a variety of human neurological age-related diseases (Naia et al., 2021). In post-menopause, luteolin decreased adipose tissue macrophage inflammation and insulin resistance (Baek et al., 2019). In addition, luteolin’s beneficial effects can also be attributed to the suppression of oxidative stress-induced cellular senescence by combating senescent phenotypes and enhancing SIRT1 expression (Zhu et al., 2021). These studies suggest that luteolin plays a critical role in promoting healthy aging and maybe increasing healthspan by modulating multiple biological mechanisms of aging.

#### 4.2.3 Icariin

Is a type of flavonoid labeled as a prenylated flavonol glycoside. This compound is widely distributed in several botanical species of the genus *Epimedium*. Icariin, including its active metabolic metabolites and semi-synthetic derivatives, has evolved to be an anti-aging molecule with stimulating effects on age-related diseases. Icariin enhanced brain function in aging rodents by increasing neuronal autophagy (Zheng et al., 2021). This ability of icariin to induce neuronal autophagy/mitophagy was *via* the AMPK/mTOR/ULK1 pathway, suggesting that it can modulate intrinsic nutrient-sensing signaling to engage the cellular trash management system effectively (Wang et al., 2021a; Zheng et al., 2021). Besides, age-related neurological disease models have shown that icariin is neuroprotective and reverses cognitive deficits in Alzheimer’s disease and Parkinson’s disease (Zhang et al., 2019b). In addition, another elegant study has demonstrated the youth-like outcomes in response to icariin treatment. In the study, aside from icariin reducing oxidative and inflammatory biomarker readouts, enhancing motor coordination and learning skills, and upregulating age-related signaling macromolecules like SIRT1/3/6 in old mice, icariin also uniquely modulated and restored beneficial microbial phenotypes in aged mice (Li et al., 2021a). This distinct feature of icariin on gut microbiota composition modulation during aging was further confirmed by fecal microbiota transfer, suggesting that besides the classical hallmarks of aging, the distorted gut microbiota composition may also account for the rapid decline in host metabolic functions, leading to accelerated biological aging. This finding adds to the complexity of targeting biological aging and suggests modulating the gut microbiota community might be another feasible option to combat age-related disorders or extend life. Besides, studies of the microbiome have also associated harmful gut microbial metabolites and disturbances within the gut flora community with cancer, osteoarthritis, cardiovascular diseases, and other aging-related diseases’ development and progression. Beyond the above, icariin was useful against aging-related testicular dysfunction, DNA damage, photoaging, etc., (Hwang et al., 2018; Li et al., 2019; Zhao et al., 2020). Icariside II, the bioactive form of icariin, extended the life of *C. elegans via* the insulin/IGF-1 signaling pathway (Cai et al., 2011). In addition, recent clinical trials and basic studies have found that icariin has a favorable effect on bone health. This implies that icariin can be used alone or in combination with other agents to slow or halt osteoporosis onset and decrease the chances of bone fractures in the elderly (Indran et al., 2016). Together, this evidence suggests a strong role for icariin in maintaining metabolic health *via* modulation of metabolic signaling and gut microbiota in aging.

#### 4.2.4 Nobiletin

Is a polymethoxylated flavonoid usually derived from citrus peels. Numerous elegant studies have swiftly identified biological targets and pharmacological mechanisms for nobiletin in the years that have passed by. Using an unbiased chemical screen, Zheng Chen’s research team and collaborators discovered nobiletin as a clock amplitude-enhancing small molecule (He et al., 2016). Many subsequent rocketing studies from the team show that the small molecule nobiletin enhances healthy aging because nobiletin prevents circadian disruption-induced age-related abnormalities by fortifying cellular bioenergetics (Nohara et al., 2019a; Mileykovskaya et al., 2020). In animal neurological disease systems, nobiletin beneficially regulated neuroinflammatory pathways and aging-related emotional disturbances, as well as regulated various clock-controlled metabolic genes, likened to insulin signaling and mitochondrial function, suggesting that nobiletin could be used in age-neurodegenerative diseases like Alzheimer’s disease and Parkinson’s disease (Braidy et al., 2017; Kim et al., 2021; Ito et al., 2022). Furthermore, several labs have revealed exquisite pharmacological activity details of nobiletin during aging and age-associated detrimental defects, which revolve around lipid homeostasis protection, antioxidation, metabolism and inflammation regulation, and cellular senescence amelioration (Nakajima et al., 2013; Nohara et al., 2019b; Shen et al., 2022). In an *in-vivo* worm model, nobiletin enhances longevity and alleviates aging-related detrimental abnormalities by increasing resistance to biologic and environmental stresses, enhancing physical performance, and scavenging reactive oxygen species (Yang et al., 2020). These studies suggest that nobiletin dampens age-related functional declines in multiple tissues and organs and may promote healthy aging.

#### 4.2.5 Curcumin

Is the prime curcuminoid abundant in *Curcuma longa* (turmeric) species. This natural compound is well-tolerated (despite some caveats on its bioavailability and result interpretation due to PAINS), and because of that, it is widely marketed and used as a dietary supplement and in cosmetics. It is one of the most studied compounds during aging and age-associated pathologies. On 27 January 2022, we searched PubMed for the number of publications involving curcumin and aging (search terms: “Curcumin” and “Aging”) and found 578 clinical, preclinical, and review articles from 1999 to 2022. From this search, many studies show that in mice, worms, yeasts, and flies, curcumin or its semi-synthetic derivatives or its combination with other nutraceuticals remarkably extend lifespan and promote healthy aging (Zhou et al., 2021a; Cheng et al., 2021). Furthermore, numerous excellent narrative and systematic reviews describe how curcumin and/or its analogs have multiple anti-aging effects (Wang et al., 2021b). In Alzheimer’s disease, for example, curcumin or its analog supplementation improves memory, inhibits tau aggregation, and reduces extracellular β-amyloid deposits in the brain, whereas, in diabetes, curcumin supplementation lowers blood glucose levels and improves insulin secretion. It suppresses inflammation and promotes vasorelaxation in rheumatoid arthritis and hypertension, respectively (Su et al., 2020; Behl et al., 2021; Mokgalaboni et al., 2021). So far, several action mechanisms for curcumin in biological aging have been proposed, including mitophagy/autophagy induction, inflammation and oxidation suppression, AMPK and SIRT1 pathway stimulation, senescence, mTOR inhibition, and mitochondrial function maintenance (Hamano et al., 2021; Zia et al., 2021; Dhapola et al., 2022). This broad range of effects of curcumin indicates that curcumin or its semi-synthetic derivatives target or bind to numerous intracellular macromolecules or modulate transcriptional responses to influence diverse aging-related pathways. Encouraged by understanding the role of curcumin supplementation on quality of life within an aging society, Sadeghian, Mehdi et al. performed a systematic review and meta-analysis of 10 randomized-control trials. They noted that curcumin administration enhances health-related quality of life despite the heterogeneity among the included studies (Sadeghian et al., 2021). In addition, in the elderly, curcumin was more effective in improving cognitive function, according to another systematic review and meta-analysis (Zhu et al., 2019). More and more human systematic review and meta-analysis studies have demonstrated the beneficial effects of curcumin on age-related detrimental effects, including lowering glucose levels (Melo et al., 2018), C-reactive protein, and high-sensitivity C-reactive protein inflammatory biomarkers (Gorabi et al., 2022), circulating interleukin six concentrations (Derosa et al., 2016), serum malondialdehyde, and enhancing superoxide dismutase activity (Qin et al., 2018). Together, curcumin can be developed as an anti-aging drug with potent efficacy in age-associated diseases, but more research needs to be done to characterize curcumin activity, considering its pharmaceutical application caveats.

#### 4.2.6 Baicalin

Is a natural flavone glycoside widely distributed in the leaves of several botanical species in the genus *Scutellaria*. From the currently available information from laboratory studies, baicalin exerts momentous beneficial medical effects necessary to combat cancer progression, neuropathologies, photodamaged skin, and other redox-related disorders. Baicalin administration activated nuclear receptor peroxisome proliferator-activated receptor-γ and modulated fork head protein O1 phosphorylation and acetylation in model aged-laboratory-adapted species (Kim et al., 2012; Lim et al., 2012). Interestingly, these molecular-induced responses by baicalin led to enhanced changes in catalase gene expression during aging and decreased age-related inflammation and reactive oxygen species production in cellular systems. The transcription factor nuclear factor-kappaB is a cellular macromolecule that is extremely sensitive to redox changes. It has also been found that the anti-oxidative effects of baicalin on age-related oxidative stress are due to the suppression of nuclear factor kappaB signaling (Kim et al., 2006). These data suggest that baicalin modulates multiple cellular pathways and post-translational modification processes to arrest age-related redox imbalance and inflammation, which are critical factors in aging biology. However, whether these responses will culminate in extending life remains to be determined. Other beneficial mechanisms of action of baicalin have been discovered, such as protecting skin cells against ultraviolet A/B-induced photoaging (Min et al., 2014; Zhang et al., 2014), inhibiting β-amyloid-induced microglial proliferation, activation, and secretion (Xiong et al., 2014), improving postoperative cognitive memory dysfunction (Zhang et al., 2020), remodeling the gut microbiota composition similar to youth-like microbiome (Liu et al., 2020), and inducing cellular senescence, suppressing gut inflammation and cancer progression (Wang et al., 2018; Wang et al., 2020). These findings reflect baicalin’s potential usefulness in preventing or suppressing causal aging determinants; hence, treatment with baicalin may be effective against age-related diseases. Because of this, several clinical studies are being conducted to test baicalin alone or in combination with other dietary products/supplements for effects on aging skin disorders (Farris et al., 2014), rheumatoid arthritis, and coronary artery disease (Hang et al., 2018).

#### 4.2.7 Resveratrol

Was discovered in the white hellebore *Veratrum grandiflorum* for the first time. It is high in various edible sources, such as grapevines (particularly *Vitis vinifera*), red wine, blueberries, blackberries, raspberries, and peanuts (Szkudelski and Szkudelska, 2015). In the Mediterranean diet, red wine is a major source of resveratrol. The average trans-resveratrol content ranged from 1.9 mg/L (8.2 μM) to 14.3 mg/L (62.7 μM). Resveratrol has been shown preclinically to help prevent various aging-related diseases, including neurodegenerative diseases, cardiovascular diseases, and diabetes (Bhullar and Hubbard, 18522015; Novelle et al., 2015). At the same time, basic studies suggest resveratrol stimulates mitochondrial function, regulates redox and inflammatory systems, and improves biomarker indices and cellular cascades, which are deemed targets for biological aging. As an example, resveratrol prevented β-amyloid-induced neurotoxicity, neuroinflammation, and oxidative stress in an Alzheimer’s disease animal model (Drygalski et al., 2018). In addition, in part, by increasing Sir2 activity and consequently increasing the cellular NAD^+^/NADH ratio, resveratrol extends life in diverse model organisms when administered late in life (Ghosh et al., 2013; Lee et al., 2016; Orlandi et al., 2017). However, in normal diet-fed mice, resveratrol did not increase lifespan, despite doing so for high-fat diet-fed mice. The discrepancy is most likely due to the fact that, under different pathophysiological conditions, the mechanism of the Sir2 orthologues could differ across the same or different organismal species (Bhullar and Hubbard, 18522015; Novelle et al., 2015). At present, besides preclinical studies, several small/mini-sampled size human randomized controlled trials involving different doses of resveratrol have been conducted on aging diseases, with mixed findings. Several studies suggest that despite the low bioavailability, resveratrol treatment is effective, safe, and well-tolerated in elderly individuals with age-related diseases and may promote healthy aging (Movahed et al., 2020; Harper et al., 2021). Moreover, resveratrol and its metabolites can permeate the blood-brain barrier (Turner et al., 2015), offering some supportive grounds for animal studies that have suggested resveratrol and its enhanced nano-formulations as potential anti-Alzheimer’s disease drugs (Li et al., 2021b). Aside from neurodegenerative diseases, resveratrol is beneficial for various diabetes-related organ injuries, including diabetic neuropathy, diabetic retinopathy, diabetes-induced liver damage, and cardiovascular diseases. In human trials, resveratrol lowered systolic blood pressure in hypertensive patients and blood glucose levels in type 2 diabetes mellitus patients (Breuss et al., 2019; Dyck et al., 2019). Nonetheless, in a pilot study of overweight older adults, 1,000 mg of resveratrol but not 300 mg elevated cardiovascular risk biomarkers (Mankowski et al., 2020) and was ineffective in adults with mitochondrial myopathy in another cross-over randomized controlled trial (Løkken et al., 2021). This means that there is a need to rationalize the doses of resveratrol that are therapeutically beneficial or detrimental, and further suggests that basic research should explore different doses of resveratrol under aged-challenged conditions. Substantial evidence shows that resveratrol-induced beneficial cellular responses during aging are *via* AMPK and SIRT1 stimulation. Because, in the absence of SIRT1, resveratrol could not activate AMPK (Price et al., 2012), and similarly, AMPK-dependent improvements in mitochondrial function and biogenesis were not observed in SIRT1 negated mice. These findings, among others, imply that SIRT1 is required for downstream AMPK activation, and resveratrol binds to SIRT1 to induce conformational activation changes necessary for stimulating the SIRT1/AMPK pathway. Indeed, the X-ray crystal structure of SIRT1 in complex with resveratrol has been solved (RSCB PDB ID: 5BTR). The structure shows that resveratrol activates SIRT1 by mediating the interaction between the 7-amino-4-methylcoumarin-containing peptide and the SIRT1 N-terminal domain and enhancing firm binding between SIRT1 and the peptide (Cao et al., 2015). However, resveratrol is not a perfect sirtuin-activating compound because of its weak SIRT1 activity. One possible explanation for this is its poor pharmacokinetics indices. Interestingly, current synthetic sirtuin-activating compounds affect aging-related diseases, and like resveratrol, they share a common mechanism for allosteric activation of SIRT1 (Hubbard et al., 2013). So far, SRT2104 and SRT1720 are the most promising sirtuin-activating compounds (Milne et al., 2007) with beneficial effects in treating aging-related diseases. This shows that, out of understanding resveratrol’s anti-aging effects, several other synthetic anti-aging agents have been obtained, paving the way for drug discoveries with potential usefulness in extending lifespan and suppressing harmful aging-dependent toxic chemicals (Hoffmann et al., 2013; Venkatasubramanian et al., 2013; Mercken et al., 2014; Mitchell et al., 2014; van der Meer et al., 2015; Bonkowski and Sinclair, 2016).

#### 4.2.8 Quercetin

Is a flavonol and is a common ingredient in most marketed dietary supplements or foods in the world. Close to what has previously been discussed for other natural products, quercetin can also prolong the median lifespan in diverse model organisms when administered late in life (Saul et al., 2008; Proshkina et al., 2016; Berkel and Cacan, 2021). Various evidence from basic studies suggests its therapeutic potential in age-related diseases such as neurodegenerative diseases, degenerative joint disorders, cardiovascular diseases, metabolic diseases, etc., (El-Sayed et al., 2021; Grewal et al., 2021; Dhanya, 2022). With quercetin, modulation of the oxidant-antioxidant system and inflammatory pathways has been linked to its capacity to enhance longevity (Williamson et al., 2018). Moreover, compelling evidence exists that quercetin, together with dasatinib, is a remarkable senolytic cocktail with promising anti-aging effects. Because this senolytic cocktail of quercetin and dasatinib effectively clears senescent cells and senescence-associated secretory phenotypes *in-vitro* and in aged humans and mice (Zhou et al., 2021b; Lin et al., 2021; Krzystyniak et al., 2022). In the elderly population, intervertebral disc degeneration is prevalent. Dasatinib and quercetin reduced degeneration and senescence markers p16^INK4a^, p19^ARF^, and senescence-associated secretory phenotype proinflammatory cytokines (Novais et al., 2021). Most importantly, human trials indicate that this senolytic drug combination may alleviate the burden of senescence-associated secretory phenotype, proinflammatory cytokines, and senescent cells in fatal idiopathic pulmonary fibrosis and diabetic kidney patients (Justice et al., 2019; Hickson et al., 2020). Overall, these studies suggest that quercetin profoundly affects cellular senescence. Therefore, quercetin drug combinations designed to inhibit cellular senescence to prevent age-related diseases should be carefully studied for any off-target effects.

#### 4.2.9 Epigallocatechin-3-Gallate

Is from green tea and is among the most investigated natural compounds. EGCG shares the property of exerting anti-aging effects. The regulation of inflammaging, lipid metabolism, autophagy, mitochondrial function, and energy metabolism is further supported by compelling evidence that treatment with EGCG in laboratory disease models prolonged lifespan and correlated with inhibition or induction of longevity genes such as SIRT1 (Yuan et al., 2020). Furthermore, EGCG has effects on an organism’s response to nutrient levels. AMPK appears implicated in most instances because EGCG has an indirect activation effect on AMPK. Also, the fact that EGCG induces autophagy further implies its ability to affect an mTOR-centered network to improve metabolic health and longevity (Brimson et al., 2021). Interestingly, under certain dose ranges of EGCG, lifespan-extending effects have been reported in laboratory-adapted mice and worms. In a small sample size clinical study, aging was found to have induced glucose-oxidative stress and advanced glycation end-product formation in the elderly, especially those who smoke. However, EGCG usage reduces this age-related pathophysiological alteration, further highlighting a positive correlation between the intake of this compound and the anti-type II diabetes effects (Bazyar et al., 2020; Wu et al., 2022). A current study has offered insights into the different dose effects of EGCG by demonstrating that 200 μM but not 1,000 μM of EGCG extend life in *C. elegans*. This was because high-dose EGCG accelerated the biological aging process *via* triggering the nuclear accumulation of DAF-16 (homolog of mammalian fork head box transcription factors class O). Nonetheless, combining high-dose EGCG with theanine reversed high-dose EGCG-induced lifespan reduction in *C. elegans* (Peng et al., 2021). Considering this current limitation, the characterization of dose-specific responses of EGCG will help us understand how EGCG targets aging-metabolic pathways while also avoiding deleterious effects on health.

#### 4.2.10 Ginsenosides

Are steroid glycosides and triterpene saponins found in *Panax ginseng*, *Panax notoginseng*, *Panax quinquefolium*, and *Panax japonicas*. Ginseng has become one of the most high-regarded nutritional components marketed in a wide range of dietary supplements because of its numerous purported benefits. Of all the available ginsenosides isolated from the genus *Panax*, anti-aging interventional research usually studies ginsenoside Rb1, Rd, Re, Rg3, and Rg1. Most of these interventional studies evidently demonstrate that these ginsenosides are candidate anti-aging drugs that could prevent accelerated tissue and cell aging (Lee et al., 2021). It is worth pointing out, however, that most of the anti-aging effects of ginsenosides were reported primarily in rodents and worms, and their broad anti-aging mechanisms usually center around their antioxidant, anti-inflammatory, anti-apoptotic, and mitochondria protective effects as well as activating nutrient-sensing response signaling, inducing autophagy, and preventing cell senescence (Wang et al., 2021c; Yu et al., 2021). Untangling the relative contributions of these mechanisms of action to accelerated aging processes. Moreover, basic studies suggest that ginsenosides confer protection in invertebrate and rodent models against age-related disorders, including cognitive and motor impairment, cardiovascular and metabolic dysfunction (Han et al., 2021; Ke et al., 2021). Despite these links, it remains uncertain whether, in healthy or elderly individuals,’ dietary supplementation of ginsenosides can slow aging. However, on the basis of these small sample size clinical studies outcomes (Chung et al., 2021; Jung et al., 2021), it is tempting to say ginsenosides or ginseng extracts might be better for increasing healthspan—rather than lifespan—effectively minimizing the period of frailty associated with aging or aging diseases at the end of life.

#### 4.2.11 Caffeine

Is a popular psychoactive natural drug found in coffee beans of the *Coffea* plant but can also be found in the seeds and leaves of several botanicals. It is a purine alkaloid with well-established central nervous system stimulatory effects. In the world today, people preferentially consume coffee and tea containing caffeine to improve their cognitive performance attention score, reduce tiredness, and among others. Daily caffeine consumption is common among older adults in most countries. Caffeine has also received attention for its role as a possible protective drug against age-dependent diseases, particularly Alzheimer’s disease and Parkinson’s disease. For example, a recent study from Australian Imaging, Biomarkers, and Lifestyle study showed that in cognitively normal older adults, coffee intake protects against Alzheimer’s disease and reduces cognitive decline. These protective effects resulted from coffee slowing cerebral Aβ-amyloid accumulation and Aβ-amyloid-facilitated oxidative stress and inflammatory processes (Gardener et al., 2021). In addition, Lebeau et al. recently reported that caffeine is protective against cardiovascular disease. Caffeine exerted this cardiovascular protective effect through the increased regulation of hepatic endoplasmic reticulum calcium levels to wedge sterol regulatory element-binding protein 2-induced proprotein convertase subtilisin/kexin type 9 expression to augment low-density lipoprotein receptor-mediated cholesterol clearance (Lebeau et al., 2022). However, short- and long-term caffeine consumption’s therapeutic or beneficial results are far from unequivocal since studies demonstrate conflicting benefits for caffeine intake on cognitive disorders and cognition (Zhou and Zhang, 2021). Even some report indicates that high caffeine consumption can exacerbate generalized anxiety disorders and left-ventricular function (Nwabuo et al., 2020) and may also elevate fall risk in older adults (Briggs et al., 2021). In yeast, coffee infusions remarkably extended their chronological lifespan. Coffee protected yeast against ROS, double DNA-strand break, and decreased metabolic activity (Czachor et al., 2020). On the other hand, in a racially and ethnically diverse prospective cohort study, caffeinated coffee, decaffeinated coffee, or caffeinated tea consumption did not influence survival to age 90 years in 14,659 older women during follow-up (Shadyab et al., 2020). In all, despite caffeine remaining at the forefront as the most consumed natural product through coffee beverages, evidence that high or low caffeine consumption promotes longevity is incompletely unknown.

**FIGURE 4:**
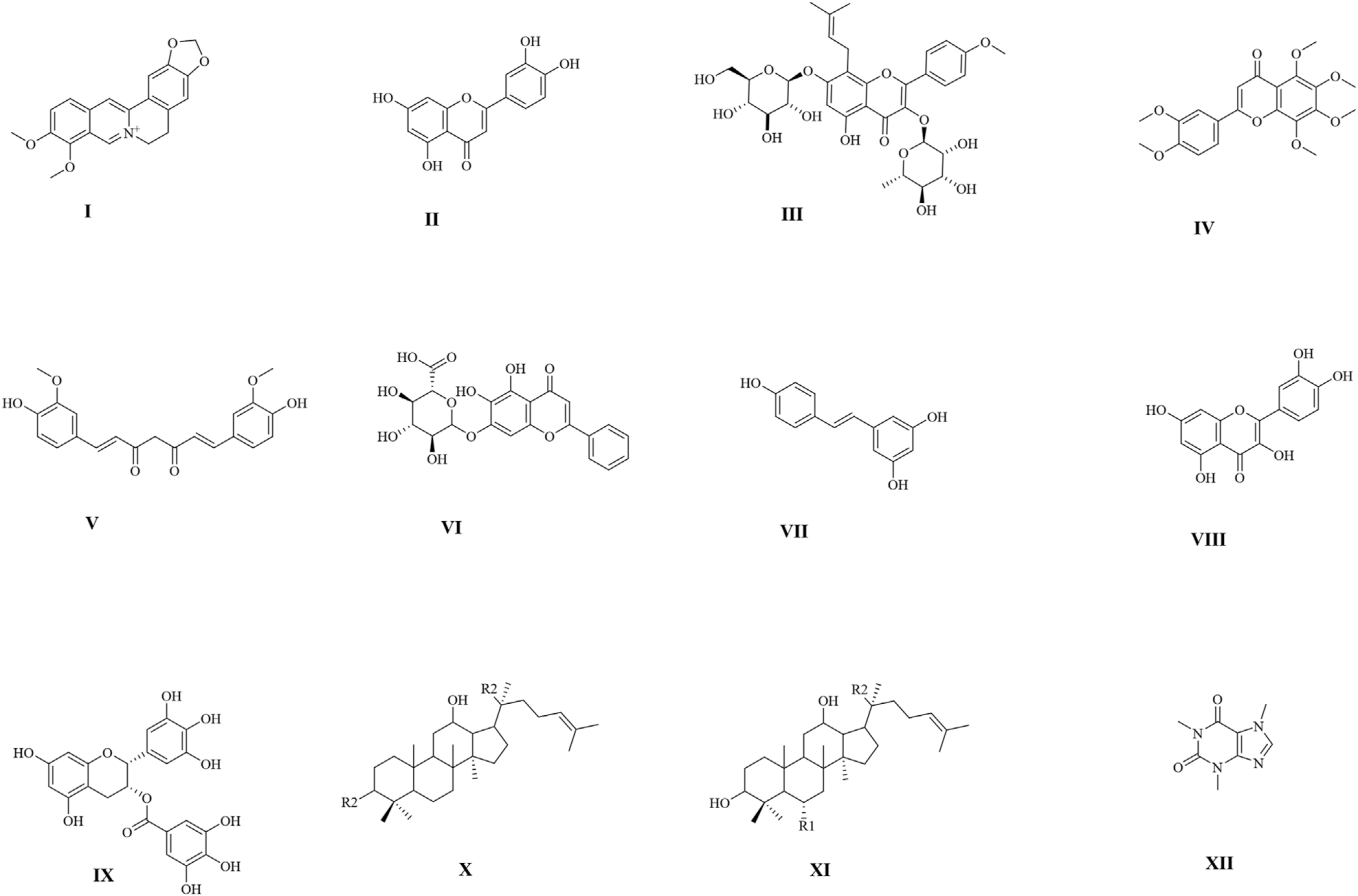
2D chemical structures of natural products with potential anti-aging properties that are considered to promote healthspan and improve aging-related diseases. (I) Berberine, (II) Luteolin, (III) Icariin, (IV) Nobiletin, (V) Curcumin, (VI) Baicalin, (VII) Resveratrol, (VIII) Quercetin, (IX) Epigallocatechin-3-gallate, (X) Ginsenoside protopanaxadiol {Ginsenoside Rb1 (R1 = -O-Glc-Glc; R2 = -O-Glc-Glc); Ginsenoside Rd (R1 = -O-Glc-Glc; R2 = -O-Glc); Ginsenoside Rb2 [R1 = -O-Glc-Glc; R2 = -O-Glc-Ara(p)]; Ginsenoside Rg3 (R1 = -O-Glc-Glc; R2 = OH)}, (XI) Ginsenoside protopanaxatriol [Ginsenoside Re (R1 = -O-Glc-Rha; R2 = -O-Glc); Ginsenoside Rf (R1 = -O-Glc-Glc; R2 = -O-Glc); Ginsenoside Rg1 (R1 = -O-Glc; R2 = -O-Glc)], and (XII) Caffeine. The 2D chemical structures were s*ketched* using ChemBioDraw software ultra 12.0 (CambridgeSoft, US).

All in all, the discussed natural products (Figure 4) have valuable outcomes on healthspan in diverse organisms, and their therapeutic effects against aging-related dysfunctions and disorders constitute an exciting and expanding field of research. However, much remains to be learned about whether all these proposed functional roles mediated by these natural products could extend life in humans. Thus, more work, particularly pushing for well-controlled, large-scale prospective international clinical trials and translational studies incorporating real-time old-age conditions and behaviors, is required to understand the efficacy of natural products in promoting healthy aging. On the premise of the molecular details of the action of natural products, it is time to explore how natural products modulate aging-linked biological receptors or genes with different efficacy.

## 5 Concluding Remarks and Outlook

In the past decades, aging has emerged as the strongest and most well-established risk factor for human disease development and major causes of death and disability. Only recently have scientists extensively expounded the biology of aging, bringing into focus the evolutionarily conserved mechanisms of aging available to control the functional declines and onset of diseases linked to aging processes. Evidently, regulatory hallmark strategies that explain the connection between aging and physiological events and lifestyle have offered a gateway for potential drug development. Herbs/botanicals, murine and terrestrial animal medicinal-derived compounds, minerals, and other dietary supplements, which are bona fide materials of nature, have been demonstrated to have anti-aging properties and are used as a start-off for discovering potent anti-aging drugs. Indeed, today, the scientific community extensively uses small-molecule natural products and dietary interventions to discover evolutionarily conserved mechanisms of aging. And they are known to modulate mitochondrial function, senescence, and nutrient-sensing metabolic signals to improve cellular aging and age-related diseases, notably metabolic diseases, cardiovascular diseases, neurodegenerative diseases, and degenerative joint disorders. However, despite the tremendous efforts, most of what we know about the therapeutic prospects of natural products and dietary supplements during cellular aging comes from basic studies utilizing native laboratory-adapted species. Thus, we cannot be sure of the extent to which these natural products and dietary supplements modulate the mechanisms of aging to reflect the treatment effectiveness of aging in outbred populations of heterogeneous environments. To better generate knowledge that could readily translate to human aging, the low-bioavailability, and off-target and steric effects issues associated with natural products must be solved. In addition, to date, human clinical trials centered on uncovering the medical potential of natural products and dietary supplements on the health of the elderly population present contradictory findings. Most of these trials usually have a small sample size, and subgroup and sensitivity analyses are mostly absent. Therefore, a push for high-quality, well-controlled, large-scale interventional trials, together with biomedical informatics and multi-omics data, is needed to delineate the sequence of real-world events that are important for understanding the effectiveness of natural products and dietary supplements in extending life. Together, these efforts will help us establish efficacy and identify novel longevity-related genes influenced by these bioactive natural products and dietary supplements. Finally, it is worth pointing out that implementing well-organized randomized controlled, double blinded clinical trials to ascertain the interventional effects and actions of dietary and natural product supplements on human aging usually faces formidable barriers. Considering the complex mechanistic effects and multiple actions of dietary and natural products, clinical trial study investigators encounter daunting decisions on selecting appropriate intervention endpoints and best-matched control groups. In addition, insufficient knowledge and understanding of clinical research and lack of funding on the side of dietary and natural product scientists also hinder the proper implementation of standardized clinical trials. Also, institutional ethical and review boards mostly have regulatory systems that favor clinical trials on synthetic drugs rather than natural supplements, whose constituents are not well-defined and characterized. It is time for ethical and institutional review boards to consider enacting well-designed interventional studies regulatory requirements that support natural supplement characteristics. In all, proffering solutions to rectify these challenges will collectively decrease the elevated levels of inferior quality randomized controlled, double-blinded clinical trials of dietary and natural product supplements on human aging and aging-related diseases.
